# The Impact of Frailty on Spine Surgery: Systematic Review on 10 years Clinical Studies

**DOI:** 10.14336/AD.2020.0904

**Published:** 2021-04-01

**Authors:** Francesca Veronesi, Veronica Borsari, Lucia Martini, Andrea Visani, Alessandro Gasbarrini, Giovanni Barbanti Brodano, Milena Fini

**Affiliations:** ^1^Complex Structure of Surgical Sciences and Technologies, IRCCS Istituto Ortopedico Rizzoli, Bologna, Italy.; ^2^Spine Surgery prevalently Oncologic and Degenerative, IRCCS Istituto Ortopedico Rizzoli, Bologna, Italy.

**Keywords:** frailty, spine diseases, frailty indexes, gender

## Abstract

Frailty is a condition characterized by a high vulnerability to low-power stressor. Frailty increases with age and is associated with higher complications and mortality. Several indexes have been used to quantify frailty. Spine diseases, both degenerative and oncologic, frequently require surgery which is related to complications and mortality. Aim of the present systematic review was to collect the most frequently used frailty indexes in clinics to predict surgical outcomes in patients affected by spine diseases, taking into account gender differences. Three databases were employed, and 29 retrospective clinical studies were included in this systematic review. The identified spine pathologies were primary and metastatic spine tumors, adult spine deformity (ASD), degenerative spine disease (DSD), cervical deformity (CD) and other pathologies that affected lumbar spine or multiple spine levels. Eleven indexes were identified: modified Frailty Index (mFI), Adult spinal deformity frailty index (ASD-FI), mFI-5, Metastatic Spinal Tumor Frailty Index (MSTFI), Fried criteria, Cervical deformity frailty index (CD-FI), Spinal tumor frailty index (STFI), Frailty Phenotype criteria (FP), Frailty Index (FI), FRAIL scale and Modified CD-FI (mCD-FI). All these indexes correlated well with minor and major postoperative complications, mortality and length of stay in hospital. Results on gender differences and frailty are still conflicting, although few studies show that women are more likely to develop frailty and more complications in the post-operative period than men. This systematic review could help the surgeon in the adoption of frailty indexes, before the operation, and in preventing complications in frail patients.

Even if frailty condition has been known for more than 30 years, the definition of the frail phenotype was first given in geriatric literature by Fried in 2001 [1] and has gained wide attention only in the last years. Frailty is a biologic syndrome characterized by a high vulnerability to low-power stressors, manifested clinically by decreased functional reserve and resilience, together with multiorgan dysfunction or multimorbidity [1]. A consensus conference in December of 2012, led by the International Association of Gerontology and Geriatrics and the World Health Organization, defined frailty as ‘‘a medical syndrome with multiple causes and contributors that is characterized by diminished strength, endurance, and reduced physiologic function that increases an individual’s vulnerability for developing increased dependency and/or death’’[2].

Several procedures have been proposed for the assessment of frailty, which rely on the measure of physical functions, as accumulation of deficits and frailty phenotype, which application depends on availability in the clinical setting, and/or self-reported items on strength, energy and weight loss [3, 4]. Moreover, specific tools have been developed in definite settings [5], thus consensus on distinctive diagnostic criteria is still missing.

It is reported that the prevalence of frailty increases with age, from 4% for ages between 65 and 69 years to 26% for older than 85 [6] and it is more frequent in females than in males [6, 7]. The worldwide occurrence of frailty varies extensively between 4% and 59% due to the heterogeneity of study populations and the use of different screening tools that consider different criteria [8, 9].

The dramatic increase in old-aged population is one of the main concerns. According to the United Nations, the proportion of global population over 65 years of age is expected to rise from 9% in 2019 to 16% by 2050 [10]. With increase in life expectancy, chronic non-communicable diseases have become prevalent together with a rising number of elderly patients affected by degenerative, traumatic, oncologic or infective pathologies.

These demographic and epidemiologic transitions have a deep impact on health care provision and economic burden. A recent study from Norway highlights that patients over 65 years represent only 15% of the population, but are responsible for almost half of the total healthcare cost [11]. Moreover, in a prospective cohort study from US, pre-frailty and frailty are associated with higher subsequent total healthcare costs in older community-dwelling men [12].

Thus, the preservation of independence in aged people and the prevention of disability are priority major challenges and frailty is becoming an increasingly important concept both for its deep impact on health outcomes and impaired quality of life.

Frailty is associated with increasing disability, hospitalization, adverse health outcomes and death [1]. A number of observational studies have also shown that frailty worsened postoperative outcomes as morbidity, mortality and length of stay (LOS) [13-15] and the severity of frailty syndrome has been reported to be directly correlated with post-surgical mortality rates and complications [16].

As frailty is correlated to general surgery outcome, it might also predict the outcome in patients undergoing spine surgery, who have been reported to record a high rate of intra- and post-operative complications [17-20]. Degenerative disorders of the spine are very frequent, with 90% of adults showing some degree of degeneration of the lumbar disk or spine by age 50 [21]. Degeneration of the spine includes a wide variety of clinical conditions, as disk degeneration, spinal stenosis, facet hypertrophy, osteophytosis, foraminal stenosis and instability, leading to back pain and/or associated neurological signs [21]. Back pain affects 15%-20% of adults each year [22] and about 17.000 new cases of spinal column injuries are reported every year in US [23]. In addition, vertebral fractures are the most frequent osteoporotic fractures among aged people, together with proximal femoral and wrist fractures [24]. Spinal metastases affect 30-70% of patients with primary tumors and can lead to spinal cord compression, pain, spinal instability and pathologic fractures [25].

Hypothesis of the present study is that frailty may play a key role in the outcome of spinal surgery and may provide a useful tool for risk prediction, facilitating the decision-making process and surgery planning in patients affected by spine disease. To the best of our knowledge, only one systematic review, on associations between frailty and spine disease, has yet been conducted [26].

The aim of this study was to systematically review 10-year clinical data regarding association between frailty and outcomes after surgery for spine disease, by identifying the most used frailty indices in spine surgery.

## MATERIALS AND METHODS

**Figure 1.**
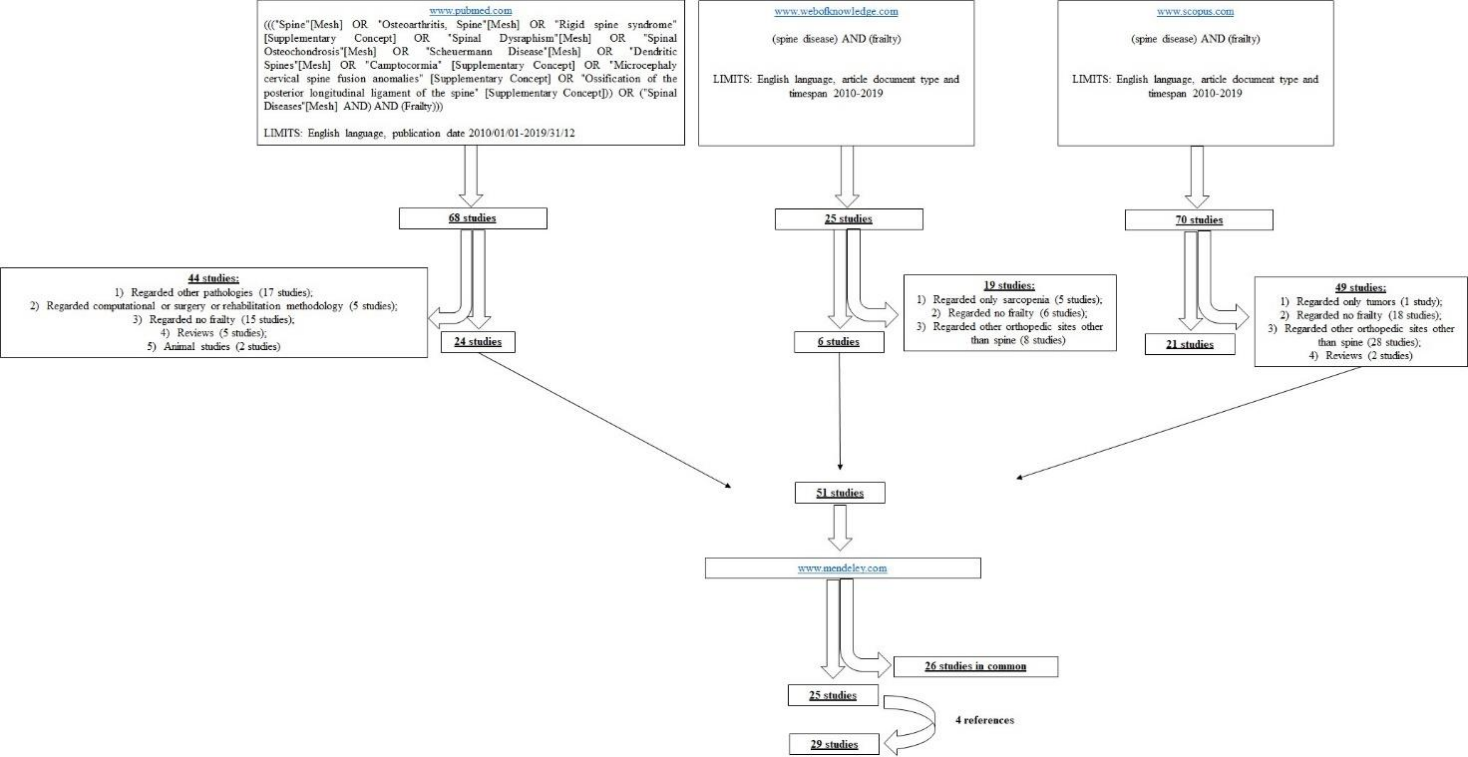
Schematic representation of the search strategy.

Three databases were employed to individuate clinical studies included in the present systematic review: www.pubmed.com, www.webofknowledge.com and www.scopus.com (Fig. 1).

In the Pubmed database the search was performed with the following meshes: ((("Spine"[Mesh] OR "Osteoarthritis, Spine"[Mesh] OR "Rigid spine syndrome" [Supplementary Concept] OR "Spinal Dysraphism"[Mesh] OR "Spinal Osteochondrosis"[Mesh] OR "Scheuermann Disease"[Mesh] OR "Dendritic Spines"[Mesh] OR "Camptocormia" [Supplementary Concept] OR "Microcephaly cervical spine fusion anomalies" [Supplementary Concept] OR "Ossification of the posterior longitudinal ligament of the spine" [Supplementary Concept])) OR ("Spinal Diseases"[Mesh] AND) AND (Frailty))). The limits were English language and publication date 2010/01/01-2019/31/12. With this search strategy, 68 studies were found.

In the Web of knowledge database, the search was performed with "(spine disease) AND (frailty)" keywords and the limits were English language, article document type and timespan 2010-2019, founding 25 studies.

In the Scopus database, "(spine disease) AND (frailty)" were also employed as keywords with the following limits: English language, article type and 2010-2019 years of publication and 70 studies were obtained.

Relevant studies were firstly screened through title and abstract by one author (FV) and the studies that did not match the argument of the review were excluded. In the first database, 24 studies were included and 44 In the first database, 24 studies were included and 44 excluded because not inherent: they regarded other pathologies (17 studies), computational or surgery or rehabilitation methodology (5 studies), not regarded frailty (15 studies) or they were reviews (5 studies) and animal studies (2 studies). In the second database, 19 studies were excluded because regarded: 1) only sarcopenia (5 studies), no frailty (6 studies) or other orthopedic sites other than spine (8 studies). In the third database, 49 studies were excluded because they regarded only tumors (1 study), no frailty (18 studies), other orthopedic sites other than spine (28 studies) or they were reviews (2 studies).

Fifty-one studies were accepted and then submitted to www.mendeley.com to eliminate duplicates, finding 26 studies in common.

The full text of the remaining 25 studies were examined by two authors (FV, VB) and the studies characteristics and results were summarized in Table 1. Finally, an additional search was performed by reading the reference lists of the 25 studies, founding further 4 studies.

So, a total of 29 clinical studies were included in the present systematic review (Fig. 1).

### Assessment of Methodological Quality

Two authors (FV, VB) independently assessed the methodological quality of the included studies with Quality in Prognosis Studies (QUIPS) tool [27] and summarized in Table 2. More precisely the tool assessed, for each study, 6 domains: 1) study participation, 2) study attrition, 3) prognostic factor measurement, 4) outcome measurement, 5) study confounding, and 6) statistical analysis and reporting. In case of disagreement, the two authors found an agreement by discussing their evaluations.

## RESULTS

As observed in Table 1, the 29 clinical studies included in this systematic review, can be divided into two groups of spinal diseases, 5 dealing with spinal tumors (primary or metastases) [28-32] and 24 dealing with other spine diseases, as adult spine deformity (ASD) [33-39], degenerative spine disease (DSD) [40-42], cervical deformity (CD) [43-45], lumbar spine diseases [46-50], diseases at different spine levels [51-55] or vertebral fractures [56]. All were retrospective clinical studies and used several different databases, with different years of recruitment, to enroll patients: Nationwide Inpatient Sample (NIS) database in 2002-2011 years [28, 29], American College of Surgeons National Surgical Quality Improvement Program (ACS-NSQIP) database in 2008-2014 [32], 2006-2012 [39, 41], 2011-2014 [46], 2010-2014 [47], 2005-2012 [48], 2012-2016 [49], 2007-2012 [51], 2006-2010 [52] or 2006-2015 [53] years, a multicenter, prospective database maintained by the International Spine Study Group (ISSG) or European Spine Study Group (ESSG) database in 2010-2014 years [34-36, 43], Spine Surgery Database of Adverse Events in 2009-2013 years [40], a multicenter database of 13 spine surgery centers across the USA in 2013-2018 years [44, 45], Spinal center of a tertiary-care teaching hospital database in 2014-2017 years [50, 56], Mount Sinai Electronic Scheduling system in 2013-2014 years [54] and not defined hospital database in which the research was carried in 2010-2015 [30], 2009-2016 [31], 2005-2015 [42], 2010-2013 [55], not specified [33, 37, 38] years.

**Table 1:** Outcomes of the 29 clinical studies performed in frail patients affected by spine pathologies.

Aim	Database employed	Spine pathology	Pts characteristics	Frailty evaluation	Results	Outcomes	Ref
Development of STFI to predict p.o complications, LOS, in-hospital mortality	NIS database (2002-2011)	Surgery for benign or malignant primary spinal neoplasms in vertebral column, sacrum and coccyx	1589 pts (28-61 yrs). 823 men, 766 women	STFI NF = 71.7% Mild frailty = 20.1% Moderate frailty = 6.0% SF = 2.2%	Mild frailty, moderate frailty and SF: ↑ all complications and LOS than NF	P.o. complications (acute respiratory distress syndrome, pleurisy, pneumothorax, pulmonary collapse, reintubation, pneumonia, PE, cardiac arrest, MI, iatrogenic stroke, acute renal failure); mortality; LOS	28
Development of MSTFI to predict perioperative complications, in-hospital mortality, LOS	NIS database (2002-2011)	Surgery for spinal metastases with a primary tumor in breast, lung, thyroid, kidney or prostate	4583 pts (54-70 yrs). 2650 men, 1931 women	MSTFI NF = 17.2%; Mild Frailty = 40.1%; Moderate Frailty = 24.7%; SF = 18.0%	Moderate frailty and SF: ↑ mortality than NF. Mild frailty, moderate frailty and SF: ↑ major complications and LOS than NF	Perioperative complications (unplanned reintubation, cardiac arrest, pneumonia, MI, PE, sepsis, acute renal failure, shock, pleurisy/pneumothorax/pulmonary collapse, adult respiratory distress syndrome, iatrogenic stroke); Mortality; LOS	29
Evaluation of mFI to predict mortality	A hospital coding database (2010-2015)	Surgery for spinal metastasis with primary tumors in prostate, unknown sites, breast, lungs, bladder, kidney, cervix, thyroid. Metastasis located in Cervical, Thoracic, Lumbar, Cervico-thoracic/Thoraco-lumbar junctions, Sacrum locations	41 pts (64±9.1 yrs). 26 men, 15 women	mFI	mFI poorly correlated with survival	Mortality	30
Evaluation of FI to predict mortality or complications	A quaternary referral center database (2009-2016)	Spinal metastases with primary tumor located in breast, lung, kidney	108 pts (35-84 yrs). 57 men, 51 women	mFI; MSTFI	mFI: correlated with complications. MSTFI: correlated with mortality	Mortality; AEs	31
Evaluation of mFI to predict mortality, major and minor complications, LOS	ACS-NSQIP database (2008-2014)	Surgery for primary and metastatic tumors in extradural, intradural extramedullary and intramedullary locations	2170 pts (57±16 yrs). 1172 men, 998 women	mFI	F: ↑ mortality and LOS than NF	Mortality; major postoperative complications (prolonged intubation of 48 hrs or more, return to the operating room, unplanned re-intubation, sepsis, venous thromboembolism, coma, stroke, cardiac arrest, septic shock, MI, surgical site/organ space infection, acute renal failure); minor complications (perioperative blood transfusion, UTI, pneumonia, renal insufficiency, wound dehiscence); LOS	32
Evaluation of ASD-FI to predict HRQoL outomes	A multicenter, prospectively collected database	Surgery for ASD with instrumented fusion of ≥ 4 levels; a minimum of 2-yrs f-up; pts with PT, PI-LL, C7SVA	332 pts (56.7±14.8 yrs). 59 men, 273 women	ASD-FI NF = 40.66%; F = 52.71%; SF = 6.63%	F: ↑ absolute changes in postoperative ODI, SF36 PCS, leg pain; the proportion of pts reaching SCB for ODI, SF-36 PCS, leg pain score than NF and SF.NF: ↑ proportion of pts reaching SCB for back pain than F and SF	SCB and change in ODI, SF-36 PCS, back pain and leg pain scores	33
Development and evaluation of ASD-FI to predict complications, LOS, reoperation rate	Multicenter, prospective database maintained by the ISSG (2010-2014)	Surgery for ASD with scoliosis (major curve≥ 20°), thoracic kyphosis ≥ 60°, PT ≥ 20°, C7SVA > 5 cm; minimum of 2 yrs of f-up	417 pts (57.67±1.13 yrs). 82 men, 335 women	ASD-FI NF = 41%; F = 39%; SF = 20%	F and SF: ↑major intraoperative and p.o. complications, any complications, LOS, junctional kyphosis than NF SF: ↑ reoperation, PJK, wound dehiscence, deep wound infection than NF	Major complications (intraoperative vascular, visceral, or neurological injury, postoperative deep infection, PE, junctional failure, similar complications); Deep wound infection rate; Wound dehiscence incidence; LOS; PJK incidence; Pseudarthrosis incidence; Reoperation rate	34
Validation and evaluation of ASD-FI to predict complications, reoperation rate, LOS	Multicenter database maintained by ESSG (2010-2014)	Surgery for ASD with scoliosis (major curve ≥ 20°), thoracic kyphosis ≥ 60°, PT ≥ 20°, C7SVA > 5 cm; age ≥ 18 yrs; minimum of 2 yrs of f-up	266 pts (54±2.03 yrs). 63 men, 203 women	ASD-FI NF = 51%; F = 34%; SF = 15%	SF: ↑ major intraoperative or p.o. complications, PJK, wound infection, reoperation, LOS than NF. F: ↑ major complications LOS than NF	Major perioperative complications (intraoperative vascular, visceral, or neurologic injury, deep wound infection, PE, junctional failure, other similar complications); LOS; Reoperation; PJK incidence; Deep wound infection rate; Surgical complications (intraoperative and immediate p.o. complications); Medical complications (stroke, DVT, PE, pneumonia, UTI)	35
Validation and evaluation of ASD-FI to predict major complications, LOS	Multicenter database maintained by ESSG	Surgery for ASD with scoliosis (major curve ≥20°), thoracic kyphosis ≥60°, PT ≥20°, or C7SVA >5 cm	267 pts (57±15 yrs). 88 men, 179 women	ASD-FI NF = 39.33%; F = 38.58%; SF = 22.10%	F: ↑ all complications than NF. SF: ↑ minor, major or all complication, LOS than NF	Major complications (intraoperative vascular, visceral, or neurologic injury, postoperative deep wound infection, PE, junctional failure, other similar complications); LOS; Overall complication incidence	36
Evaluation of the treatment status for Frailty to predict complications	Multicenter database of one institute	Surgery for ASD with scoliosis (major curve ≥ 20°), C7SVA ≥ 5 cm, PT ≥ 25°; 21 yrs; ≥ 5 fused vertebral levels, segmental pedicle screw fixation from the upper-instrumented vertebra to the lower instrumented vertebra; minimum of 2-yrs of f-up	240 pts (58.4±16.7 yrs). 19 men, 221 women	mFI NF = 59%; PF = 34%; F = 7%; G = 72%; PC = 28%	R: ↓ perioperative complications, 2 yrs overall complications, p.o. C7SVA; ↑ incidence of C-D2, C-D3 complication, SRS22 function than G and PC	Intraoperative and p.o. complications (surgical complications, surgical-site infection, other infection, excessive bleeding, delirium, cardiopulmonary, gastrointestinal, or renal diseases)	37
Evaluation of mFI-5 and mFI-11 to predict severe complications	Multicenter database of one institute	Surgery for ASD with scoliosis (major curve ≥ 30°), C7SVA ≥ 5 cm, PT ≥ 25°; ≥ 21 yrs; ≥ 5 fused vertebral levels, segmental pedicle screw fixation from the upper-instrumented vertebral to the lower-instrumented vertebral level; minimum of 2-yrs of f-up	281 pts (54.4±18.7 yrs)	mFI-5 mFI NF = 66%; PF = 22%; F = 12%	mFI-5 and mFI: excellent concordance across ASD surgery. mFI F: ↑ total complications, perioperative complications, implant-related complications, severe complications. mFI-5 F: ↑ severe complications. mFI-5 and mFI-11: strong predictive ability for severe complications	Major complications (all p.o. major complications, surgical-site infection, other infection, excessive bleeding, delirium, cardiopulmonary, gastrointestinal and renal diseases); severe complications (Clavien-Dindo grade 3, reoperation, deterioration of motor function at discharge, new motor deficit)	38
Evaluation of mFI to predict p.o. complications, mortality	ACS-NSQIP database (2005-2012)	Surgery for ASD with spinal fusion for deformity; long spinal fusion	1001 pts (59±14 yrs). 460 men, 541 women	mFI NF = 38.86%; PF = 58.14%; F = 3%	F: ↑ mortality, blood transfusion, PE/DVT, any p.o. complications, reoperation than NF	P.o. complications (pneumonia, sepsis, DVT, PE, woundcomplication, deep infection, CNS complication, sepsis/septic shock, cardiac arrest, acute renal failure, UTI, reoperation); Mortality occurring within 30 days	39
Evaluation of mFI to predict p.o. complications, LOS, discharge to a facility, in-hospital mortality	Spine Surgery Database of Adverse Events (2009-2013)	Primary DSD with spondylolisthesis, lumbar stenosis and disc herniation at thoracolumbar spine (T9-S1) level	102 pts (68-78 yrs). 51 men, 51 women	mFI NF = 59.8%; PF = 20.6%; F = 19.6%	mFI not associated with incidence of p.o. complications. F: ↑ risk of mortality than NF	Any perioperative AEs (intraoperative and p.o. complications); LOS; p.o. discharge to a facility; In-hospital mortality	40
Evaluation of mFI to predict p.o. complications, LOS, discharge disposition, mortality	ACS-NSQIP database (2006-2012)	Elective or semielective surgery for DSD with procedural related to the spine	52,671 pts (56.1±14.5 yrs). 27389 men, 25282 women	mFI NF = 46%; PF = 50%; F = 4%	F: ↑ major complication, reoperation for p.o. infection, LOS, discharge to a new facility, 30-day mortality than NF	Major complications (Clavien IV complications); LOS; Discharge to a facility that was not home; mortality within 30 days of surgery	41
Evaluation of the relationship between mFI and BMI	Not specified (2005-2015)	Surgery for DSD at cervical, thoracic and lumbar levels	1970 pts (58.1±5.91 yrs). 1045 men, 925 women	mFI NF = 42.39%; PF = 54.57%; F = 3.05% BMI underweight = <18.5; normal weight = 18.5-25; overweight = 25.0-29.9; Obese = >30.0	mFI: positive correlation with complications and negative correlation with BMI. Underweight: ↑ prefrailty and frailty. Obese: ↑ frailty. Underweight, Obese, PF and F: ↑ p.o. complications. Underweight/normal weight+PF/F, overweight+F and obese+NF/F: ↑ p.o. complications	Complications (any deviation from the normal postoperative course, requiring pharmacological treatment, blood transfusions or total parenteral nutrition, requiring radiological, endoscopic, or surgical interventions, life-threatening complications requiring ICU management, death)	42
Evaluation of CD-FI to predict preoperative risk, complications, LOS, discharge disposition	Multicenter, prospective database maintained by ISSG (2009-2015)	Surgery for CD: Cervical scoliosis (major angle ≥10°) and cervical kyphosis (major angle >10°); minimum 1-yr f-up	61 pts (61±2.7 yrs). 24 men, 37 women	CD-FI. NF = 27.9%; F = 55.7%; SF = 16.4%	SF: ↑ major complications, medical complications than NF	Major complications (intraoperative vascular, visceral, or neurologic injury, postoperative deep infection, PE, junctional failure); LOS; Discharge disposition; Medical/surgical complications (most intraoperative complications and immediate postoperative complications related to surgical technique/error, stroke, DVT, PE, pneumonia, UTI)	43
Evaluation of mCD-FI to predict p.o. clinical outcomes, complications, HRQoL, mortality	Prospectively collected, multicenter database (2013-2017) of 13 spine surgery centers across the USA	Surgery for CD: Cervical kyphosis (major angle >10°), cervical scoliosis (major angle <10°), C7SVA > 40mm or CBVA >25°	121 pts (61.47±9.8 yrs). 48 men, 73 women	mCD-FI. NF = 47.9%; F = 46.3%; SF = 5.8%	SF: ↑ overall comorbidity burden, depression, pulmonary disease than NF. F: ↑ vascular complication, superficial surgical site infection, deterioration patient-reported measures of neck pain, neck disability, and overall HRQoL, LOS than NF. SF: ↑ cardiac arrest, mortality, deterioration in patient-reported measures of neck pain, neck disability, and overall HRQoL, LOS than F and NF	LOS; Complications; HRQoL scores	44
Evaluation of CD-FI to predict p.o. complications	A prospective, multicenter database (2013-2018) of 13 spine surgery centers across USA	Surgery for CD: cervical kyphosis (major angle > 10°), C7SVA > 40 mm, TS-CL > 10° or CBVA > 25°; minimum 1-yr follow-up	138 pts (61.0 yrs). 53 men, 85 women	CD-FI	F: ↑ minor and major complications than NF	Perioperative complications	45
Evaluation of ASA, mCCI and mFI to predict p.o. complications	ACS-NSQIP database (2011-2014)	Surgery for PLF or PLIF	16,495 pts (60±13.5 yrs). 7357 men, 9138 women	mFI NF = 39.2%; PF = 58.9%; F = 1.9%	mFI and ASA: ↑ discriminative ability of any, severe and minor complications, LOS, infectious complications, discharge to higher-level care than mCCI. ASA: The most predictive comorbidity index	Severe complications (coma, cardiac arrest, death, DVT, MI, postoperative intubation, PE, return to the operating room, sepsis, stroke); Minor complications (acute kidney injury, anemia requiring transfusion, pneumonia, surgical site infection, UTI, wound dehiscence); Any complications (major or minor AEs); Infectious complications (pneumonia, sepsis, surgical site infection, UTI, wound dehiscence); LOS; Discharge to higher level of care	46
Evaluation of mFI to predict mortality, serious and overall complications	ACS-NSQIP database (2010-2014)	Surgery for ALIF	3920 pts (not reported). Not reported	mFI NF = 51.66%; PF = 47.09%; F = 1.25%	F: ↑ any complications, pulmonary complications than NF	Complications (death, pulmonary, renal, CNS, wound and cardiac complications, venous thromboembolism, UTI, sepsis, graft failure, blood transfusions); Return to the OR; LOS	47
Evaluation of mFI to predict p.o. complications, mortality	ACS-NSQIP database (2005-2012)	Surgery for lumbar spinal fusion procedures (PLF, PLIF, TLF, TLIF)	6094 pts (60±13.9 yrs). 2742 men, 3352 women	mFI NF = 37.18%; PF = 56.71%; SF = 6.10%	F: ↑ mortality, reoperation, LOS, unplanned readmission, several p.o. complications, pulmonary, renal, PE/DVT, sepsis, UTI, blood transfusion, wound complications	P.o. complications (pneumonia, sepsis, DVT, PE, wound complication, deep infection, CNS complication, sepsis/septic shock, cardiac arrest, acute renal failure, UTI); Mortality occurring within 30 days; Reoperation; Unplanned reoperation; Readmission; LOS	48
Evaluation of mFI-5 to predict 30-day p.o. surgical and medical complications, readmissions, non-home discharge and mortality	ACS-NSQIP database (2012-2016)	Surgery for elective PLFs for lumbar spinal stenosis, spondylolisthesis, degenerative disc disease, spondylosis	23,516 pts (≥ 18 yrs). 10764 men, 12752 women	mFI-5 NF = 38.11%; PF = 42.80%; F = 19.10%	PF = ↑ any complications, medical complications, 30-day readmissions, non-home discharge than NF. F = ↑ any complications, superficial and deep SSI, unplanned reoperation, medical complications (pneumonia, unplanned intubation, postoperative vent use, progressive renal insufficiency, acute renal failure, UTI, CVA/stroke, MI, bleeding transfusions, sepsis, septic shock), 30-day readmissions, non-home discharge than NF and PF	Any complications; Superficial SSI; Deep SSI; Organ/space SSI; Wound dehiscence; Unplanned reoperations; Medical complications (pneumonia, unplanned intubation, postoperative ventilator use, progressive renal insufficiency, acute renal failure, UTI, stroke, MI, bleeding requiring transfusion, sepsis and septic shock); 30-day readmissions; Mortality; Non-home discharge	49
Evaluation of frailty in LSS	Spinal center of a tertiary-care teaching hospital database (2014-2017)	LSS with a stenotic lesion in the lumbar spine	142 pts (72.1±6.9 yrs). 42 men, 100 women	Fried criteria NF = 11.97%; PF = 46.48%; F = 41.55%	F: ↑ disability; ↓ quality of life than R and PF	ODI, EQ-5D	50
Evaluation of frailty to predict perioperative morbidity and mortality	ACS-NSQIP database (2007-2012)	Surgery for spinal decompression with or without fusion or VP/KP, for thoracic fractures with or without SCI (T1-T6, T7-T12), lumbar fracture with or without cauda equine injury	303 pts (66.55±15.5 yrs). 138 men, 165 women	mFI	F: ↑ complications, 30-day mortality than NF	Perioperative complication (30-day mortality, intraoperative events, acute renal failure, ventilator use for over 48 h, cerebrovascular accident or stroke, MI, cardiac arrest, PE, sepsis, septic shock, coma for over 24 h, unplanned re-intubation); Operative time	51
Evaluation of mFI to predict p.o. morbidity and mortality	ACS-NSQIP database (2006-2010)	Lumbar laminectomy and discectomy, lumbar fusion, anterior cervical decompression, anterior cervical fusion, cervical 360° fusion, cervical laminectomy and fusion, thoracic decompression and fusion	18294 pts (not specified). 9513 men, 8781 women	mFI	F: ↑ at least 1 infection, mortality, surgical site infections, Clavien IV complications than NF	P.o. complications (wound infection, any infection, Clavien IV complications); 30-day mortality	52
Evaluation of mFI-5 to predict comorbidities and p.o. complications	ACS-NSQIP database (2006-2015)	Kyphoplasty for vertebral, lumbar or thoracic augmentation, percutaneous vertebral or lumbar augmentation	2465 pts (74 yrs). 735 men, 1730 women	mFI-5 NF = 26%; PF = 46.6%; F = 29.4%	F: ↑ at least 1 complication, readmission rate, LOS, discharged to a location other than home than NF	Complications (cardiac, pulmonary, wound, infection, hematology, renal); Other complications (Stroke/cerebrovascular incident, need for ventilator >48 hours, septic shock, sepsis, UTI); 30-day readmission; 30-day reoperation; LOS; Adverse hospital discharge	53
Evaluation of frailty to predict p.o. functional recovery and cognition	Mount Sinai Electronic Scheduling system (2013-2014)	Surgery at cervical and lumbar levels; ASA status I-III	100 pts (71 yrs). 63 men, 37 women	FRAIL scale NF = 26%; PF = 56%; F = 18%	R and PF: ↑ cognitive recovery at 3 mo after surgery than F. PF: ↓ functional recovery than F and R at 3 mo	Cognitive recovery; ADL	54
Evaluation of FP and FI to predict p.o. complications, LOS, discharge to PAC and 30-day hospital readmission	SAGES prospective cohort study (2010-2013)	Elective surgery: lumbar, cervical or sacral laminectomy	122 pts (76.8±5.2 yrs). 165 men, 250 women	FI NF = 21%; PF = 38%; F = 41%. FP NF = 11%; PF = 54%; F = 35%	Moderate concordance between FP and FI. F and PF for FI and FP: ↑ at least one adverse outcome than R. PF for FP: ↑ discharge to PAC, complications than R. F for FP: ↑ LOS, discharge to PAC, complications than R. PF and F for FI: ↑ discharge to PAC, LOS than R	P.o. medical and surgical complications; LOS; Discharge to PAC; Readmission within less than 30 days	55
Evaluation of the association between frailty and OVCF	Single center of a tertiary-care teaching hospital (2014-2017)	Old vertebral compression fracture (T7-L5) caused by a minor trauma at least 6 mo prior	59 pts (73.1±6.2 yrs). 7 men, 49 women	Fried criteria NF = 17.9%; PF = 39.3%; F = 42.9%	F: ↑ ODI; ↓ EQ-5D-5L than NF and PF. PF: ↑ ODI; ↓ EQ-5D-5L than NF. ≥3 vertebral fractures = ↑ F	ODI; EQ-5D-5L	56

ACS-NSQIP = American College of Surgeons National Surgical Quality Improvement Program; ADL = activities of daily living; AEs = adverse events; ALIF = anterior lumbar interbody fusion; ASA = American Society of Anesthesiologists; ASD = Adult Spinal Deformity; ASD-FI = Adult Spinal Deformity Frailty Index; BMI = body mass index; C7SVA = C7 sagittal vertical axis; CBVA = chin-brow vertical angle; CD = cervical deformity; CD-FI = Cervical deformity frailty index; CNS = central nervous system; CVA = cerebrovascular accident; DSD = degenerative spine disease; DVT = deep vein thrombosis; EQ-5D = EuroQol 5-dimension; ESSG = European Spine Study Group; F = frailty; FI = frailty index; FP = frailty phenotype; f-up = follow-up; G = good frailty control; HRQoL = Health related quality of life; ISSG = International Spine Study Group; LOS = length of stay; LSS = lumbar spinal stenosis; mCCI = modified Charlson Comorbidity Index; mCD-FI = modified cervical deformity frailty index; mFI = modified Frailty Index; MI = myocardial infarction; MSTFI = Metastatic Spinal Tumor Frailty Index; NF = not frailty; NIS = Nationwide Inpatient Sample; ODI = Oswestry Disability Index; OR = operative room; OVCF = osteoporotic vertebral compression fracture; p.o. = post-operative; PAC = postacute institutional care; PC = poor frailty control; PE = pulmonary embolism; PF = pre-frailty; PI-LL = pelvic incidence-lumbar lordosis; PJK = proximal junctional kyphosis; PLF = posterior lumbar fusion; PLIF = posterior lumbar interbody fusion; pts = patients; R = robust; SAGES = Successful Aging after Elective Surgery; SCB = substantial clinical benefit; SCI = Spinal Cord Injury; SF = severe frailty; SF36 PCS = 36-Item Short Physical Component Summary; SRS22 = Schwab-Scoliosis Research Society; SSI = surgical site infection; STFI = Spinal tumor frailty index; TLF = transforaminal lumbar fusion; TLIF = transforaminal lumbar interbody fusion; TS-CL = thoracic slope-cervical lordosis; UTI = urinary tract infections; VP/KP = vertebroplasty/kyphoplasty; yrs = years

### Assessment of Methodological Quality

Risks of bias assessments for each study were indicated in Table 2. Most of the studies showed an overall risk of bias low or moderate (n = 23 studies, 79,3%). Only a fraction of the studies (n = 3 studies (10,3%) [29, 30, 52] had a high risk, due to the lack of information for at least one aspect of the study attrition item [29, 30] or in analysis items, showing no statistical analysis [52].

All studies showed a low outcome measurement item because all studies had well described outcome measurement with a clear definition of the outcome, valid and reliable outcome measurements and the same method and setting of outcome measurement for all study participants. In addition, for all studies the Confounding Measurement and Account item was always moderate because the observed effect of the prognostic factors on outcome may be distorted by another factor related to the outcome.

### Spine tumors

Five studies regarded spine benign or malignant primary tumors or metastatic ones [28-32].

More precisely, patients underwent surgery for benign or malignant neoplasms in vertebral column, sacrum and coccyx [28], spinal metastasis of primary tumors located in breast [29], lungs [29-31], thyroid [29, 30], kidney [29-31], prostate [29, 30], bladder [30] or cervix [30] and one study described both primary or metastatic tumors allocated in extradural, intradural extramedullary and intramedullary sites [32].

### Frailty assessment in spine tumor studies

Three different frailty indices were used for the identification and evaluation of frailty in patients affected by primary or metastatic spine tumors (Table 3), all of them are based on the accumulation of deficit model suggested by Rockwood.

**Table 2:** QUIPS tool for assessing risk of bias in the clinical studies.

Ref	Study Participation	Study Attrition	Prognostic Factor Measurement	Outcome Measurement	Confounding Measurement and Account	Analysis
28	Low	Moderate	Low	Low	Moderate	Low
29	Moderate	High	Moderate	Low	Moderate	Low
30	Low	High	Low	Low	Moderate	Moderate
31	Low	Moderate	Low	Low	Moderate	Low
32	Moderate	Moderate	Low	Low	Moderate	Low
33	Low	Moderate	Low	Low	Moderate	Low
34	Moderate	Moderate	Moderate	Low	Moderate	Low
35	Moderate	Moderate	Moderate	Low	Moderate	Low
36	Moderate	Moderate	Moderate	Low	Moderate	Low
37	Low	Moderate	Moderate	Low	Moderate	Low
38	Low	Low	Low	Low	Moderate	Low
39	Low	Low	Low	Low	Moderate	Low
40	Low	Low	Low	Low	Moderate	Low
41	Low	Low	Low	Low	Moderate	Low
42	Moderate	Moderate	Low	Low	Moderate	Low
43	Moderate	Moderate	Moderate	Low	Moderate	Low
44	Low	Moderate	Moderate	Low	Moderate	Low
45	Low	Moderate	Moderate	Low	Moderate	Low
46	Low	Moderate	Low	Low	Moderate	Low
47	Low	Moderate	Low	Low	Moderate	Low
48	Low	Moderate	Low	Low	Moderate	Low
49	Low	Moderate	Moderate	Low	Moderate	Low
50	Low	Moderate	Moderate	Low	Moderate	Low
51	Moderate	Moderate	Moderate	Low	Moderate	Low
52	Moderate	Moderate	Moderate	Low	Moderate	High
53	Low	Low	Low	Low	Moderate	Low
54	Low	Low	Low	Low	Moderate	Moderate
55	Low	Low	Low	Low	Moderate	Low
56	Low	Low	Low	Low	Moderate	Low

Spinal Tumor Frailty Index (STFI) [28] and Metastatic Spinal Tumor Frailty Index (MSTFI) [29, 31] were respectively used in benign or malignant primary spine tumors [28] and in spinal metastases with a primary tumor located in breast, lungs, thyroid, kidney or prostate [29, 31]. Both indices grouped patients into 4 frailty categories: no frailty (0), mild frailty (1), moderate frailty (2) and severe frailty (≥3).

Modified Frailty Index (mFI) was employed in patients affected by spinal metastasis with a primary tumors in prostate, unknown sites, breast, lung, bladder, kidney, cervix, thyroid [30, 31] or primary and metastatic spine tumors in extradural, intradural extramedullary and intramedullary locations [32]. The cut-off for not frailty is 0, that for pre-frailty is 0-0.21 and that for frailty is ≥ 0.27.

### Results in spine tumor studies

Mild, moderate and severe frailty significantly increased all complications and LOS in hospital in 1589 patients with age between 28 and 61 years [28]. Similarly, moderate and severe frailty significantly increased mortality, while mild, moderate and severe frailty were associated with major complications and LOS in 4583 patients (age 54-70 years) [29].

In 41 [30], 108 [31] and 2170 [32] patients, with a mean age of 60 years, one group of authors did not find correlation between frailty status and mortality after surgery [30], while other authors showed that frail patients had higher mortality and LOS than not frail ones [32]. MSTFI was also compared with mFI, underling that mFI correlated with complications, while MSTFI with mortality [31].

### Other spine diseases

Twenty-five clinical studies regarded patients who underwent surgery for ASD (7/25 studies) [33-39], DSD (3/25 studies) [40-42], CD (3/25 studies) [43-45], lumbar spine disease (5/25 studies) [46-50] and diseases involving different spine levels (5/25 studies) [51-55], that comprised also patients with osteoporotic vertebral fractures (1/25 study) [56].

**Table 3:** Frailty indices employed in the 29 clinical studies included in the systematic review.

	Frailty Index name	Frailty index acronym	Items	Frailty scale	Ref.
Accumulation of deficit model	Spinal tumor frailty index	STFI	1) Anemia; 2) congestive heart failure; 3) chronic obstructive pulmonary disease; 4) coagulopathy; 5) electrolyte abnormalities; 6) pulmonary circulation disorders; 7) renal failure; 8) malnutrition; 9) pathologic fractures	NF: 0; Mild Frailty: 1; Moderate frailty: 2; SF: ≥ 3	28
	Metastatic Spinal Tumor Frailty Index	MSTFI	1) Anemia; 2) Chronic lung disease; 3) Coagulopathy; 4) Electrolyte abnormalities; 5) Pulmonary circulation disorders; 6) Renal failure; 7) Malnutrition; 8) Emergent/urgent case; 9) Anterior or combined surgical approach	NF: 0; Mild Frailty: 1; Moderate Frailty: 2; SF: ≥ 3	29, 31
	Modified Frailty Index	mFI	1) Non-independent functional status; 2) history of diabetes mellitus; 3) history of chronic obstructive pulmonary disease; 4) history of congestive heart failure; 5) history of myocardial infarction; 6) history of percutaneous coronary intervention, cardiac surgery, or angina; 7) hypertension requiring the use of medication; 8) peripheral vascular disease or rest pain; 9) impaired sensorium; 10) transient ischemic attack or cerebrovascular accident w/o residual deficit; 11) cerebrovascular accident w/o deficit	NF: 0; PF: 0-0.21 F: ≥ 0.27	30, 31, 32, 37, 38-42, 46-48, 51, 52
	Adult spinal deformity frailty index	ASD-FI	1)Health deficits documented by physician: >3 medical problems; BMI <18.5 or >30 kg/m2; Cancer; Cardiac disease; Currently on disability; Depression; Diabetes; Hypertension; Liver disease; Lung disease; Osteoporosis; Peripheral vascular disease; Previous blood clot (DVT/PE/stroke); Smoking status. 2)Health deficits patient-reported: Bladder incontinence; Bowel incontinence; Deteriorating health this yr; Difficulty climbing 1 flight of stairs; Difficulty driving a car; Difficulty getting dressed; Difficulty getting in/out of bed; Difficulty sleeping >6 hrs; Difficulty walking 100 yards; Difficulty w/o light activity; Feeling downhearted/depressed most of the time; Feeling tired most of the time; Feeling worn out most of the time; General health: fair/poor; Inability to bathe w/o assistance; Inability to cheer up often; Inability to do normal work/schoolwork/housework; Inability to lift heavy objects; Inability to travel >1 hr; Inability to walk w/o assistive device; Leg weakness; Loss of balance; Not in excellent health; Personal care dependency; Restricted activity level; Restricted social life	NF: < 0.3; F: 0.3-0.5; SF: > 0.5	33-36
	Modified Frailty Index 5	mFI-5	1) history of severe chronic obstructive pulmonary disease; 2) congestive heart failure within 30 days before surgery; 3) functional health status prior to surgery (independent versus partially or totally dependent); 4) hypertension requiring medication; 5) diabetes mellitus with oral agents or insulin	NF: 0; PF: 1; F: ≥ 2	38, 49, 53
	Cervical deformity frailty index	CD-FI	1)Health deficits documented by physician: >3 Medical problems; Anxiety; BMI <18.5 or >30; Cancer; Cardiac disease; Cerebrovascular disease; Currently receiving disability benefits; Dementia; Depression; Diabetes; Liver disease; Lung disease; Neuromuscular disease; Osteoporosis; Pancreatic disease; Rheumatoid arthritis; Smoker; Vascular disease; Venous disease; Unsteady gait. 2)Health deficits patient-reported: Bladder incontinence; Bowel incontinence; Difficulty driving; Difficulty getting dressed; Difficulty reading; Difficulty sleeping >6 h; Difficulty walking without assistive device; Feeling anxious or depressed most of the time; Feeling tired most of the time; Feeling weak most of the time; Feeling worn out/exhausted most of the time; General health <50; Inability to concentrate; Inability to do normal work/schoolwork/housework; Inability to engage in normal recreational activity; Inability to lift heavy objects; Inability to perform normal activities; Inability to walk; Leg weakness; Personal care dependency	NF: < 0.2; F: 0.2-0.4; SF: > 0.4	43, 45
	Modified cervical deformity frailty index	mCD-FI	1)Lung disease; 2) BMI <18.5 kg/m2 or >30 km/m2; 3) Diabetes; 4) Depression; 5) Liver disease; 6) Rheumatoid arthritis; 7) Venous disease; 8) Unsteady gait; 9) Bladder incontinence; 10) Leg weakness; 11) Comorbidities; 12) Anxiety; 13) Bowel incontinence; 14) Difficulty sleeping >6 h; 15) Inability to walk	NF: < 0.3; F: 0.3-0.5; SF: >0.5	44
	Frailty Index	FI	1) Help Bathing; 2) Help Dressing; 3) Help getting in/out of Chair; 4) Help Walking around house; 5) Help Eating; 6) Help Grooming; 7) Help Using Toilet; 8) Help up/down Stairs; 9) Help lifting 10 lbs; 10) Help Shopping; 11) Help with Housework; 12) Help with meal Preparations; 13) Help taking Medication; 14) Help with Finances; 15) Lost more than 10 lbs in last year; 16) Self Rating of Health; 17) How Health has changed in last year; 18) Stayed in Bed at least half the day due to health (in last month); 19) Cut down on Usual Activity (in last month); 20) Walk outside; 21) Feel Everything is an Effort; 22) Feel Depressed; 23) Feel Happy; 24) Feel Lonely; 25) Have Trouble getting going; 26) High blood pressure; 27) Heart attack; 28) CHF; 29) Stroke; 30) Cancer; 31) Diabetes; 32) Arthritis; 33) Chronic Lung Disease; 34) MMSE; 35) Peak Flow; 36) Shoulder Strength; 37) BMI; 38) Grip Strength; 39) Usual Pace; 40) Rapid Pace	NF: 0.15; PF: 0.15-0.24; F: ≥ 0.25	55
Phenotypic model		FRAIL scale	1)fatigue over the past 4 months; 2) ability to climb a flight of stairs unassisted; 3) ability to walk two blocks unassisted; 4) medical comorbidities; 5) loss of weight	NF: 0; PF:1-2; F: 3-5	54
		FRIED criteria	1) weight loss; 2) exhaustion; 3) physical inactivity; 4) slowness; 5) handgrip strength	NF: 0; PF:1-2; F: ≥ 3	50, 56
	Frailty Phenotype criteria	FP criteria	1) Slow gait (3-m timed walk); 2) Weakness (grip strength); 3) Low activity (energy expenditure); 4) Involuntary weight loss; 5) Exhaustion	NF: 0; PF: 1-2; F: ≥ 3	55

### Frailty assessment in spine disease studies

As observed in Table 3, 9 frailty indices were employed to stratify patients affected by different spine diseases that needed surgery. mFI was yet employed in tumor section. Some of them are included in the accumulation of deficit model suggested by Rockwood (ASD-FI, mFI, mFI-5, CD-FI, mCD-FI), while the others follow the phenotypic model suggested by Fried (Fried criteria, FRAIL scale, and FP).

Adult Spinal Deformity Frailty Index (ASD-FI) [33-36] stratified patients affected by ASD, into not frail (< 0.3), frail (0.3-0.5) and severe frail (> 0.5) ones.

mFI and its trunked version, mFI-5, were employed in patients affected by ASD [37-39], DSD [40-42], or subjected to posterior lumbar fusion (PLF) or posterior lumbar interbody fusion (PLIF) [46, 48], anterior lumbar interbody fusion (ALIF) [43, 47], thoracic fractures [51] or diseases at different spine levels [52], lumbar stenosis, spondylolisthesis, degenerative disc disease and spondylosis [49] and patients subjected to Kyphoplasty [53].

CD-FI [43, 45] divided patients, affected by CD, into not frail (< 0.2), frail (0.2-0.4) and severe frail (> 0.4). Also, CD-FI had a trunked version, mCD-FI [44] with little differences, from CD-FI: not frail (< 0.3), frail (0.3-0.5) and severe frail (> 0.5).

Fried criteria [50, 56] and FRAIL scale [54] stratified patients, affected by stenotic lesions of the lumbar spine, vertebral fractures [56] and elective surgery at cervical and lumbar levels [54] into not frail (0), pre-frail (1-2) and frail (≥ 3) ones.

Finally, FI and FP were compared in one study [55], in patients that underwent to elective surgery at cervical and lumbar levels. FP divided patients into not frail (0), pre-frail (1-2) and frail (≥ 3) ones, while FI into not frail (0.15), pre-frail (0.15-0.24) and frail (≥ 0.25) ones.

### Results in spine disease studies

In 332 [33], 417 [34], 266 [35], 267 [36], 240 [37], 281 [38] and 1001 [39] patients of a mean age of 57 years and affected by ASD, frail and severe frail patients showed significantly higher intraoperative and postoperative complications, any complications, reoperation, proximal junctional kyphosis (PJK), wound dehiscence, deep wound infection, LOS and junctional kyphosis than not frail ones [34-36].

Frailty significantly increased mortality rate, blood transfusion, pulmonary embolism/deep vein thrombosis (PE/DVT), any postoperative complications and reoperation rate than not frailty [39]. It was also observed that frailty significantly increased the absolute changes in postoperative Oswestry Disability Index (ODI), 36-Item Short Physical Component Summary (SF36 PCS), leg pain and the proportion of patients that reached substantial clinical benefit for ODI, SF-36 PCS, leg pain score also in comparison to frailty and severe frailty [33].

Prefrail and frail patients in good control group or poorly controlled group experienced more perioperative complications and postoperative C7 sagittal vertical axis (C7SVA) than frail patients [37]. The control group of frailty was defined as treatment following the appropriate guidelines for each mFI factor [37]. Making a comparison between the classic mFI and the truncated form mFI-5 items it was observed an excellent concordance, especially in the prediction of complications. The classic mFI was able to well correlate with total complications, perioperative complications, implant-related complications, while mFI-5 with severe complications [38].

In patients affected by DSD, frailty significantly increased risk of mortality, major complication, reoperation for postsurgical infection, LOS and discharge to a new facility [40, 41], even if one study did not find association with the incidence of postoperative complications [40]. A correlation between frailty and body mass index (BMI) in the prediction of postoperative complications was founded [42]. Underweight, obesity, prefrailty and frailty separately increased postoperative complications and underweight significantly increased prefrailty and frailty, while obesity only frailty. In addition, underweight and normal weight associated with pre frailty or frailty, overweight associated with frailty and obese associated with not frailty or frailty increased postoperative complications [42]. The number of patients were 12 [40], 52671 [41] and 1970 [42] with a mean age of 62 years.

An amount of 61 [43] and 121 [44] severe frail patients with a mean age of 61 years, with CD, showed higher major complication, medical complications, overall comorbidity burden, depression and pulmonary disease in comparison to not frail ones [43, 44] and cardiac arrest, mortality, deterioration in patient-reported measures of neck pain, neck disability and overall Health related quality of life (HRQoL) and LOS more than not frail and frail patients [44]. In addition, 121 [44] and 138 [45] frail patients with a mean age of 61 years significantly increased vascular complication, superficial surgical site infection, deterioration patient-reported measures of neck pain, neck disability, and overall HRQoL, LOS, minor and major complications than not frail ones [44, 45].

In lumbar spine diseases, frailty was significantly associated with increase in any complications, pulmonary complications, mortality, reoperation, LOS, unplanned readmission, several postoperative complications, PE/DVT, sepsis, urinary tract infections (UTI), blood transfusion and wound complications than not frailty [47, 48]. In comparison with American Society of Anesthesiologists (ASA) score, mFI was less predictive of postoperative comorbidities, even if both were associated with severe complications, LOS, infectious complications and discharge to higher-level care [46]. Frail patients increased any complications, disability, superficial and deep Surgical site infection (SSI), unplanned reoperation, medical complications (pneumonia, unplanned intubation, postoperative vent use, progressive renal insufficiency, acute renal failure, UTI, cerebrovascular accident (CVA)/stroke, myocardial infarction (MI), bleeding transfusions, sepsis, septic shock), 30-day readmissions, nonhome discharge, disability and poor HQoL more than not frail or pre-frail ones [49, 50].

The patients enrolled were 16495 [46], 3920 [47], 6094 [48], 23516 [49], and 142 [50] at a mean age of 64 years.

In 303 [51], 18294 [52] and 2465 [53] patients (mean age of 70 years), that underwent different spine level surgery, frailty significantly increased complication rate, 30-day mortality, at least 1 infection and surgical site infections [51, 52], at least 1 complication, readmission rate, LOS and discharged to a location other than home [53] than not frailty.

One hundred frail patients of 71 years showed significantly high reduction in cognitive recovery at 3 months after surgery than not frail and pre-frail ones, and pre-frail patients showed significant higher reduction in functional recovery 3 months after surgery not only in comparison to not frail, but also than frail patients [54].

Frailty index (FI) and frailty phenotype (FP) were compared, showing a moderate concordance because both indices observed that adverse outcomes significantly increased with frailty and pre-frailty, but FI was associated with increased discharge to postacute institutional care (PAC) and LOS in pre-frail and frail patients, while FP was associated with discharge to PAC and complications in pre-frailty and increased discharge to PAC, complications and LOS in frailty [55]. The patients were 122 with a mean age of 77 years.

Finally, frailty significantly increased ODI and decreased EuroQol 5-dimension questionnaire (EQ-5D-5L) than not frailty and pre-frailty, calculated with Fried criteria in 59 patients with 73 years mean age [56].

**Table 4:** Studies addressing association between gender and frailty on spine pathologies.

Frailty Index	Spine Disease	Outcome	Conclusions	Ref
ASD-FI	ASD	Female = (88.1%) NF; (79.4%) F; (68.2%) SF *p* = 0.028: NF *Vs* F and SF; F *Vs* SF	Frailty severity decreased with female sex	33
mCD-FI	CD	Female = (48.3%) NF; (71.4%) F; (71.4%) SF *p* = 0.034: F and SF *Vs* NF	Frailty severity increased with female sex	44
mFI	DSD	Male = (50.8%) NF; (53%) PF; (60.1%) F *p* < 0.0005: F *Vs* NF and PF. Male sex: ↑ major complications, LOS, discharge disposition than female sex *p* < 0.0005 Male *Vs* female	Frailty severity increased with male sex	41

### Gender and frailty

Among the 29 studies, 3 addressed gender and its association with frailty or morbidity associated with some spine pathologies, such as ASD [33], DSD [41] and CD [44] (Table 4).

Regarding female gender, 2 studies evaluated frailty through ASD-FI [33] and mCD [44], showing contrasting results. More precisely, in 1 study the percentage of not frail women was significantly higher than that of frail and severe frail (p = 0.028) [33], while in the second study the opposite was observed: the percentage of not frail women was significantly lower than frail and severe frail ones [44]. In women affected by ASD, the severity of frailty decreased [33], while in those affected by CD, the severity of frailty increased [44].

As for male gender, in men affected by DSD, the severity of frailty, evaluated with mFI, increased [41], because the percentage of frail men was significantly higher than not frail or pre frail ones (p < 0.0005). In addition, men showed higher major complications, LOS and discharge deposition than women (p < 0.0005) [40].

## DISCUSSION

The present systematic review summarizes the most frequent frailty indices used in literature to predict surgical outcomes in frail patients undergoing surgery for several different spine diseases: primary or metastatic tumors [28-32], ASD [33-39], DSD [40-42], CD [43-45], lumbar spine pathologies [46-50] or multilevel spine ones [51-56]. In this review, frailty indices are also correlated with the most common postoperative complications.

Frailty is defined as a reduction in physiological function, but it is separated from the concept of aging, leading to the conclusion that the physiological aging is distinct from the chronological one [57]. Frailty increases the health vulnerability and deterioration, especially in elderly and several different tolls are actually used to measure frailty.

Frailty prevalence increases with age and is correlated with disability, admission to hospital and mortality and it is observed an increase of its prevalence in patients undergoing surgery than the other patients (42%-50% Vs 4%-10%) [58]. Frailty is a predictor of morbidity and mortality, more than age in elderly patients undergoing general surgery. Before surgery, the measurement of frailty and the stratification of patients become important for predicting complications, even if no consensus is found on which is the best frailty tool [59].

As the population ages, spine surgery needs to grow, to improve neurologic adverse events and pain. Since spine surgery is associated with complications or even mortality, it is important to select patients at higher risk before surgery, also with a view to reducing the costs of the health system [60, 61].

For these reasons, the present systematic review collects the most employed frailty indices able to evaluate the association of frailty and spine surgery outcomes for different spine diseases, to give an indication on which to use in the different cases before surgery.

Frailty indices are composed by items that comprise presence of some concomitant pathologies, the functional status, mood, cognitive capacity and health deficits measured by physician or by the patients. The cut-off that stratify the patients are obtained by dividing the number of the positive items for the total number of the items.

According to the results of this review, frailty indices based on accumulation of deficit model suggested by Rockwood (STFI, MSTFI, FI, mFI, ASD-FI, CD-FI, mFI-5 and mCD-FI, are ) were employed in most of the studies [28-49, 51-53, 55] in comparison to the phenotypic model suggested by Fried (FRAIL scale, FRIED criteria and FP criteria) [50, 54-56].

In this review one group of spine pathologies, requiring surgery, is primary or metastatic tumors. The spinal metastasis incidence is between 30 and 70% among patients with primary tumors and 10% of the metastases undergoes surgery [62]. Three frailty indices are used: STFI [45] in primary tumors and MSTFI [46, 48] and mFI [47, 48, 51] in metastatic ones. STFI and MSTFI are correlated with perioperative complications and MSTFI with mortality. Both indices are composed of 9 items that regard the presence of cardiovascular, respiratory, urinary and musculoskeletal system disorders and malnutrition. “Pathologic fracture” and “congestive heart failure” items of STFI are replaced by “emergent/urgent case” and “anterior or combined surgical approach” items in MSTFI.

mFI is the most famous and the most frequently used frailty index in literature also in other pathologies and it consists of 11 variables, that regard non-independent functional status and the history of concomitant pathologies. In spine tumors it is observed that frailty, measured with mFI, is correlated with mortality and complications, even if only one study does not find a correlation between mFI and survival [30].

mFI and its truncated form mFI-5, characterized by 5 items, are also in common in other spine pathologies that required surgery, identified in this review, including ASD [37-39], DSD [40-42] or patients undergoing PLF [46, 48, 49], PLIF [46, 48], ALIF [47], transforaminal lumbar fusion (TLF) [48], transforaminal lumbar interbody fusion (TLIF) [48], thoracic fractures [51], lumbar, cervical or thoracic procedures [52] and kyphoplasty [53].

ASD and other spine pathologies are usually associated with postoperative or perioperative complications, due to the invasiveness of surgical procedures, including large dissection, multilevel fusion or osteotomy [63].

In these cases, besides mFI, other indices are used as ASD-FI, CD-FI, Fried criteria, FRAIL scale, FI and FP criteria.

ASD-FI, composed of 40 items, is divided into health deficits documented by physician (14 items) and health deficits patient-reported ones (26 items) and is employed in patients affected by ASD [33-36]. Similar frailty index is CD-FI, employed in patients suffered of CD [43, 45], composed by 40 items, health deficits documented by physician (20 items) and health deficits patient-reported ones (20 items). As mFI, also CD-FI possesses its truncated form, that comprises 15 items instead of 40 ones, that take into consideration the presence of diseases, BMI, weakness, anxiety and difficulty in sleeping or in walking [44].

FRIED criteria [50, 56] and FRAIL scale [54] are easier than the other ones because they are composed by 5 items and regard prevalently subjective functional performances: weight loss, exhaustion, physical inactivity, slowness and handgrip strength. The differences between the two indices is that FRAIL scale substitutes physical inactivity, slowness and handgrip strength with ability to climb a flight of stairs unassisted, ability to walk two blocks unassisted and medical comorbidities. FP, employed only by one study [55], takes into consideration similar items to FRIED and FRAIL indices. The same study that employed FP, compared it with FI (40 items) [55], that regards the need for help in carrying out daily actions, weight loss, mood and presence of pathologies.

All these frailty indices correlate well with perioperative or postoperative complications, mortality or overall survival, LOS, discharge to a facility that is not home. The most evaluated complications regard the respiratory (acute respiratory distress syndrome, pleurisy, pneumothorax, pulmonary collapse, reintubation, pneumonia, pulmonary embolism), cardiac (cardiac arrest, myocardial infarction, iatrogenic stroke) and urinary (acute renal failure) systems, sepsis, shock, intraoperative vascular, visceral or neurological injury, deep infection, prolonged intubation, return to the operating room, unplanned re-intubation, venous thromboembolism, coma, perioperative blood transfusion, urinary tract infection, wound dehiscence, pseudoarthrosis incidence, excessive bleeding and delirium.

One of the most awaited discussions in the scientific community concerns the method by which complications are collected. Unfortunately, many groups use the most disparate and personalized methods to collect complications, making a comparison difficult and often underestimating the real percentage of the problem. Chen et al. show that another fundamental point in collecting complications lies in the people who collect them. Surgeons underestimate the problem, while a group of external observers, not involved in surgery, seems the best people to collect complications in the right measure [64]. The two most frequent grading methods for complications in vertebral and orthopedic surgery are the Spine Adverse Event Severity System (SAVES) and Clavein-Dindo one. The first allows systematic prospective collection of postoperative adverse events in spinal surgery and is divided into 14 intraoperative and 22 postoperative events, the second is based on the therapy used to correct a specific complication and is divided into 7 grades [65, 66]. Only few studies employed these grading methods for complications, one study the SAVES [31], and 8 studies the Clavein-Dindo [37, 38, 41, 42, 49, 51-53].

The different definitions and classifications of complications by different investigators make difficult to compare studies, dividing complications into major (that lead to reoperation or permanent deficits) and minor ones. So, a limit of the present review could be the different methods used to record complications and the group that evaluates them. For this reason, frailty probably generates more complications than those published.

Most of the studies of the present review validate and evaluate one frailty index for each study. However, in 3 cases, the same study compared two frailty indices [31, 38, 55]. In patients with spinal metastases of primary tumors located in breast, lung and kidney, mFI and MSTFI were compared, showing that mFI correlated with postoperative complications, while MSTFI with mortality [31]. mFI and mFI-5 showed an excellent correlation across ASD surgery and were strong predictive for severe complications, but mFI correlated with total, perioperative and severe complications, while mFI-5 with severe complications [38]. Finally, a moderate accordance between FP and FI indices was observed. FP correlated with discharge to PAC and complications, while FI with discharge to PAC and LOS [55].

The patients included in the studies varied from a minimum of 41 [30] to a maximum of 52671 [41] and were both men and women, but a prevalence of studies enrolled more women than men [33-37, 39, 43-46, 48-51, 53, 55, 56]. This seems to presage that, between the two genders, there is a prevalence of women who are frail compared to men. Still now, few studies identify gender differences in frailty. Three frailty indices are able to discriminate between males and females, but the results are discordant because frailty severity seems to decrease [33, 41] or increase [44] with female gender. More precisely, ASD-FI and mFI show that frailty severity decreases in women affected by ASD and DSD, respectively, and that men shows higher major complications, LOS and discharge disposition than women [33, 41]. Conversely, mCD-FI indicates that frailty severity increases more in women than in men affected by CD [44].

However, since only 3 studies dealt with gender difference, with heterogeneity in the study design, study participants, and spine pathologies, it was difficult to draw any significant conclusion regarding this theme.

Frailty is a prevalent age condition, but in this review 14/29 studies considered also patients younger than 60 years [28, 29, 31, 32-39, 41, 42,49]. This reinforces the idea that physiological aging is distinct from the chronological one and that frailty indices can be applied at any age in pathologies of the spine.

## Conclusion

In summary, this systematic review identified 11 frailty indices that correlated well with complications of spine surgery outcomes, also with severe complications. Even if there is no consensus on which is best, mFI is the most employed and the most adaptable to all spine pathologies. Indeed, it is employed in metastatic tumors [30-32], ASD [37-39], DSD [40-42], lumbar pathologies [46-49] or multi-level pathologies [51-53]. In decreasing order of frequency the other indices are ASD-FI, exclusively in ASD pathology [33-36], CD-FI only in CD pathology [43-45], MTSFI in metastatic tumors [29, 31], Fried criteria in lumbar spine pathologies [50] and vertebral fractures [56], FRAIL scale in cervical and lumbar pathologies [54], FP and FI in lumbar, cervical and sacral pathologies [55] and STFI in primary spine tumors [28].

Because it is one of the most complete indices, having 11 items that concern the functional status and the history of concomitant pathologies. At the same time, it is also less complex than other indices that may contain up to 40 items.

Clarity has not yet been made regarding the relationship between the frailty level and gender, even if a worsening of frailty is prevalently observed in women.

Given the paucity of the studies regarding the comparison between different frailty indices in the same study and of the studies regarding the evaluation of gender in frailty, it will be mandatory to deepen these comparisons in future studies.
